# Nutritional and Rheological Characteristics of Composite Flour Substituted with Baobab (*Adansonia digitata* L.) Pulp Flour for Cake Manufacturing and Organoleptic Properties of Their Prepared Cakes

**DOI:** 10.3390/foods10040716

**Published:** 2021-03-27

**Authors:** Hassan Barakat

**Affiliations:** 1 Department of Food Science and Human Nutrition, College of Agriculture and Veterinary Medicine, Qassim University, Buraydah 51452, Saudi Arabia; haa.mohamed@qu.edu.sa or hassan.barakat@fagr.bu.edu.eg; Tel.: +966-547-141-277; 2 Food Science Department, Faculty of Agriculture, Benha University, Moshtohor, Qaliuobia 13736, Egypt

**Keywords:** functional and fortified cakes, *Adansonia digitata*, nutritional properties, amino acids, organoleptic properties

## Abstract

Revalorization of *Adansonia digitata* L. “Baobab” pulp flour (BPF) to produce a notorious and functional cake in the current study was assessed. Wheat flour (WF 72%) was partially substituted by BPF at 5, 10, and 15% to prepare composite flour (WF + BPF) for potential cake manufacturing. Approximate chemical composition, macro- and microelements content, amino acids (AAs), total phenolic content (TPC), and antioxidant activity (AOA) of partially substituted composite flour (WF + BPF) were determined. The rheological properties of the composite flours were assessed using MIXOLAB. Moreover, an organoleptic evaluation of the baked cakes was performed with 20 trained panelists. The substitution with BPF significantly increased the total ash and crude fiber content in composite flour in a level-dependent manner, while moisture, crude fat, crude protein, available carbohydrates contents, and energy values were not significantly changed. Interestingly, macroelements such as Ca, K, and P were significantly increased, while Na was significantly decreased, whereas Mg content was not significantly changed. Similarly, microelements such as Zn, Fe, and Cu increased with the increase of BPF substitution. Significant increases in TPC and AOA were found by increasing the substitution with BPF. The biological value (BV), essential amino acid index (EAAI), protein efficiency ratio (PER), as well as essential amino acids (EAAs) requirement index (RI) were positively improved in WF + BPF. Adding BPF up to 10% not only improved the water absorption, α-amylase activity, and viscosity, but also caused a slight weakness in the gluten network, to produce a composite flour suitable for cake making. Conclusively, this study revealed that fortification with BPF up to 5–10% improved the nutritional quality without adverse effects on technological, and organoleptic characteristics and providing economic, commercial, and health benefits.

## 1. Introduction

Cakes are the highest consumed bakery products, despite being particular goods, but they are mostly used in festivals and happy celebrations [1,2,3]. Cake is usually manufactured from soft wheat flour (WF), which is deficient in fibers and phytochemicals when processed at higher extraction [4,5]. In addition, the amino acids (AAs) profile of WF is deficient in some vital and essential amino acids (EAAs) such as lysine, threonine, and methionine [6]. *Adansonia digitata* L. named also “Baobab”, belongs to the Malvaceae family and is a majestic tree revered for its medicinal and nutritional values [7]. It is tolerant to high temperatures and long spans of drought and is grown for its sour fruit and leaves. Seeds, leaves, roots, flowers, fruit pulp, and bark of *A. digitata* are edible and incorporated in various diets [7,8,9]. The fruit consists of pulp and large seeds embedded in a dry acidic pulp, which is incorporated in food preparation and drinks [10]. The fruit pulp has a very high vitamin C content, almost ten times that of oranges, and contains sugars but no starch. Moreover, the fruit pulp is rich in pectin [9]. Several parts of this tree have gained much interest for their antioxidant and anti-inflammatory properties, which are frequently included in traditional medicine [11]. Proximate composition and concentration of minerals in pulp and seed were determined [12]. Considerable content of fiber, K, Ca, Mg, Na, P, Fe, and Zn was observed. Hyacinthe et al. [13] confirmed that *A. digitata* has valuable content of vitamin B_1_, B_2_, and minerals. Seeds are a good source of energy, protein, and fat. Amino acid analyses revealed high glutamic and aspartic acid content, with the sulfur-containing amino acids being the most limited amino acid [9,14]. The fatty acid profile showed that oleic and linoleic were the major unsaturated fatty acids, whereas palmitic was the major saturated acid [9]. Hydroxycinnamic acid glycosides, iridoid glycosides, and phenylethanoid glycosides were found to be the main components in Baobab fruit pulp [15]. HPLC analysis revealed that *A. digitata* has appreciable levels of flavonoids and phenolic acids, including catechin, epicatechin, rutin, quercitrin, quercetin, kaempferol, luteolin, gallic, chlorogenic, caffeic, and ellagic acids [16]. Recently, Sokeng et al. [17] indicated that the profiling of primary and secondary metabolites of Baobab fruit and leaves addresses the limited knowledge of Baobab’s chemical composition and helps to support and clarify the increasing evidence of its nutritional and biological properties and to provide suggestions on the potential use of Baobab fruit and leaves in food, pharmaceutical, and cosmetic products. In fruit pulp, 46 phytochemicals belonging mainly to proanthocyanidins, phenolic acids, flavonols, and saponins were identified [18]. The promising potential of Baobab fruit shells (BFSs) as a good source of phenolic compounds that can be used in food and pharmaceutical applications was demonstrated by Ismail et al. [19].

Metabolic syndrome comprises a group of risk aspects for many pathological circumstances, including hyperglycemia, obesity, hyperlipidemia, and hypertension. The traditional information, taxonomic description, medicinal properties, and important nutritional value of *A. digitata* were summarized [7]. Fruit pulp and leaf extracts of *A. digitata* improved the metabolism of carbohydrates and lipids [16,20,21]. Its aqueous extract demonstrated antimetabolic syndrome potential, remarked as weight loss, anti-inflammatory, hypolipidemic, hypoglycaemic, renal, hepatic, and cardio-protective activities [22,23] as well as analgesic effect [24]. Adeoye et al. [25] confirmed that apigenin and quercetin as secondary metabolites of ethylacetate partitioned fraction of *A. digitata* stem bark possess antimalarial activity, in silico. A positive effect of the use of Baobab to alleviate hunger, possibly having a positive effect on the maintenance of weight, was indicated [26]. Hanafy et al. [27] found a significant protective effect of *A. digitata* extract against hepatotoxicity through amelioration of lipid peroxidation. In rats with predominantly normal myocardial structure and no inflammatory cell infiltration, Baobab pulp flour (as 200 mg per day of BPF) resulted in a cardioprotective effect against induced oxidative stress [28]. *A. digitata* is rich in flavonoids with superior antioxidant activity [29], which may not ultimately limit the bioavailability of carotenoids [30]. Present minerals and phytochemicals in Baobab root tubers suggest their nutritional and medicinal potential [31]. The fruit pulp of *A. digitata* is rich in procyanidins and flavonol glycosides, with tiliroside as the major constituent which provides a promising source of health-promoting substances [32]. Adding Baobab seed extract (BSE) to beef patties improved the lipid stability and keeping quality as a result of increasing the antioxidant and antimicrobial activities which extended the shelf-life [33]. Mixing Baobab milk and fermented Baobab/acha (African grain) flour provided more nutrients than the Baobab or the acha flour alone, which can be incorporated as a nutritious and economic ingredient into diets of the lower-income group [34].

Due to its current use as emergency food during food shortages and the relatively healthy and stable rejuvenating populations, a recent study showed great potential for Baobab to become an important part of the diet [35]. Moreover, BPF flour could potentially become an interesting improver and structuring agent for gluten-free products [36]. However, despite the literature showing great potentialities related to the use of BPF flours, the effects of BPF flour substitutions on dough rheology and cake characteristics need to be carefully examined. Particular attention needs to be paid to the milling process (both for wheat and for BPF) [37], dough rheology [4,5], and final cake characteristics [4,5]. Moreover, to the best of the authors’ knowledge, the literature review mainly highlighted the substitution of WF with interesting alternative flours like insects [38,39], legumes [40], and other by-products [41], but only a few studies considered substitutions with BPF flours, which need further investigation, thus motivating this work. Therefore, this study aimed to investigate the effects of WF substitution with 5%, 10%, and 15% of BPF on the chemical composition, minerals, and amino acids content, total phenolic content (TPC), and antioxidant activity (AOA); rheological parameters of different substituted flour and sensory evaluation of baked cakes were performed as well.

## 2. Materials and Methods

### 2.1. Baobab Pulp Flour and Cake Processing

The obtained Baobab (*A. digitata* L.) fruits in mature and normal dried status (sun drying) were transferred immediately to the analytical lab. The fruits of Baobab were cleaned, shells of the fruits were opened, and then coated seeds with dry pulp were removed. Subsequently, seeds were ground gently by a grinder (Severin, type Km 3871, Mecklenburg, Germany) to separate the pulp from the seeds and passed across a 60-mesh sieve to get a fine homogenous powder [42]. The BPF was directly filled in dark-glass jars, then kept at −18 ± 1 °C until use. The WF 72% was partially substituted by BPF at 0, 5, 10, and 15% before cake manufacturing. Formulations of substituted WF at various substitution levels by BPF and additional ingredients are illustrated in Table 1. The manufacturing method of the cake was carried out by applying method No. 10-90.01 according to the American Association of Cereal Chemists (A.A.C.C.) [43] using similar ingredients of Khalifa et al. [44].

### 2.2. Approximate Chemical Composition 

The WF 72% and various substituted WF with BPF were subjected to the chemical composition analysis (moisture, crude protein, crude fat, ash, and crude fiber) according to A.A.C.C. [42]. The available carbohydrate content by difference on a fresh weight basis and total energy value for all macronutrients (kcal 100 g^−1^) were calculated according to the Food and Agriculture Organization (FAO) [45].

### 2.3. Determination of Minerals 

The mineral content (including sodium, calcium, and potassium) was determined in WF, BPF, and substituted WF with BPF using flame-photometry, while magnesium, iron, copper, and zinc content was determined by atomic-absorption spectroscopy according to the Association of Official Analytical Chemists (A.O.A.C.) [46]. A typical colorimetric procedure was used for phosphorus, as described by Borah et al. [47].

### 2.4. Total Phenolic Content and Antioxidant Activity

The TPC of methanolic extracts of WF, BPF, and substituted WF with BPF was determined by applying the Folin–Ciocalteau protocol [48]. The TPC was expressed as milligram gallic acid equivalents per gram of sample (mg GAE g^−1^ dry weight, DW). The antioxidant activity (AOA) of the various substituted flours was assessed using DPPH^●^ assay [49]. The absorbance at 515 nm was recorded, and results were expressed as mg of Trolox equivalents (TE) per gram of dry weight (mg TE g^−1^ DW). The radical-scavenging activity (RSA) of WF, BPF, and substituted WF with BPF against the stable ABTS (2,2′-azino-bis(3-ethylbenzothiazoline-6-sulfonic acid)) radical cation was assessed using the method of Barakat and Rohn [50]. The Trolox calibration curve was plotted on the basic of the percentage of ABTS radical-cation-scavenging activity. The final findings were expressed as micromoles of Trolox Equivalents (TE) per gram (mol of TE g^−1^ DW).

### 2.5. Determination of Amino Acids

The amino acids profile was carried out on the precipitated protein from defatted WF, BPF, and substituted WF with BPF after hydrolysis by 6.0 N HCl for 24 h at 110 °C in evacuated ampoules. Quantitative determination of amino acids was carried out by Amino acids analyzer (Biochrom 30 Series, Biochrom Limited, Cambridge, England) and using built-in Biochrom 30 Series control software (software for data collection and processing, 32 bit graphical software) according to A.O.A.C. [51]. The predicted biological value (BV) [52] and predicted protein efficiency ratio (PER) [53], and amino acid score [54] were estimated, in vitro.

### 2.6. Rheological Properties

The effect of BPF substitution with 5, 10, and 15% on WF thermo-mechanical and rheological properties was determined. The analysis was performed in triplicate using the MIXOLABiapparatus (20 AV MARCELLINiBERTHELOT, CHOPIAN, France) according to Dubat [55].

### 2.7. Organoleptic Properties

Evaluation of the organoleptic properties of various substituted cakes was carried out in morning sessions (11:00–13:00 h) by 20 well-trained panelists according to A.A.C.C [43]. The panelists used mineral water to rinse out the mouth among samples. Cake samples were left to cool at room temperature for 1 h after baking, then cut with a sharp knife and distributed in disposable plates (10 × 20 cm^2^) after coded using random numbers and subjected to panel test as 4 samples to each panelist at once. The score was distributed as 40: crumb cells, 30: texture, 10: crust color, 10: odor, 10: taste, and 100: overall acceptability.

### 2.8. Statistical Analysis

All analyses were carried out in triplicates, except for amino acids, performed in duplicates. Statistical analysis was carried out using the SPSS program v. 23, (IBM, USA) under a significance level of 0.05. The one-way ANOVA using the percentage of WF substitution levels, that is, 0, 5, 10, and 15%, was applied, then multiple comparisons were carried out with Tukey’s multiple-range test corresponding to Steel et al. [56].

## 3. Results

### 3.1. Chemical Composition of WF, BPF, and Substituted WF with BPF

The proximate chemical composition of WF, BPF, and their mixtures was determined; data are shown in Table 2. The moisture, ash, and fiber content in BPF was significantly higher (*p* < 0.05) than in WF. No significant difference was found between BPF and WF in crude protein content, as well as in related energy values. Available carbohydrate and crude fat content was significantly higher in WF than in BPF. The partial substitution of WF with BPF up to 15% did not significantly alter the moisture, crude protein, crude fat, and available carbohydrate content but significantly increased ash and fiber content.

### 3.2. Mineral Content of WF, BPF, and Substituted WF with BPF

Minerals analysis of BPF used in this experiment showed it to contain a lower content of sodium (36.02 mg 100 g^−1^) and higher content of macroelements, e.g., Ca (237.03 mg 100 g^−1^), K (987.51 mg 100 g^−1^), P (124.08 mg 100 g^−1^), and Mg (102.01 mg 100 g^−1^), when compared with WF; Table 3. Microelements Zn, Fe, and Cu showed higher content in BPF than in WF. Substituting WF with BPF up to 15% significantly increased the content of Ca, K, P, Mg, Zn, Fe, and Cu as well as significantly decreased Na in a level-dependent manner. The increasing rate of minerals in the WF + 15% BPF formula was 83.10, 73.52, 6.61, 2.11, 30.30, 95.87, and 64.29%, respectively. As recorded, incorporation of BPF valuably increased Ca, K, Zn, Fe, and Cu. On the contrary, partial substitution of WF with 5, 10, and 15% of BPF decreased Na content by 4.84, 7.87, and 13.10%, respectively.

### 3.3. Total Phenolic Content and Antioxidant Capacity of WF, BPF and Substituted WF with BPF

The TPC and relative AOA using DPPH and ABTS assays of all formulated flours were investigated; data are shown in Table 4. The TPC and AOA were significantly higher in BPF than in WF. Probably, increasing of substitution level up to 15% resulted in increasing the content of TPC and relevant AOA. The increasing rate was 12.7, 25.7, and 38.4% for substituting levels of 5, 10, and 15%, respectively. Accordingly, the AOA increased with increasing the substitution level, and the highest values were recorded in WF + 15% BPF for both DPPH and ABTS radicals. Incorporating the BPF into flour formulas of the cake improved the AOA by 17.9, 36.0, and 53.9% in WF + 5% BPF, WF + 10% BPF, and WF + 15% BPF, respectively. Similarly, assessing the AOA using ABTS radicals indicated a 21.8, 43.7, and 65.4% increase when 5, 10, and 15% of WF were substituted by BPF.

### 3.4. Amino acids profile of WF, BPF, and substituted WF with BPF

The amino acid profiles of WF, BPF, and substituted WF with BPF up to 15% are reported in Table 5. Certainly, amino acid compositional data just comprise the first step in every food protein’s nutritional evaluation. Data obtained showed that eighteen amino acids were determined in BPF, WF, and substituted samples. The total EAAs and total nonessential amino acids (NEAAs) of BPF exhibited higher content than their content in WF. It has been found that both EAAs and NEAAs have increased with increasing BPF levels. Substituting WF with 5, 10, and 15% BPF increased total EAAs and total NEAAs by 1.03, 2.41, and 3.08% and 0.41, 1.25, and 1.33%, respectively. 

Consequently, partial substitution of WF with BPF resulted in an improvement in AAs profile, which enriched EAAs better than it did NEAAs. However, comparing the content of individual EAAs and NEAAs between BPF and WF revealed that each EAA and NEAA content in BPF was higher than its content in WF, except methionine, proline, and tyrosine were higher in WF than in BPF. Remarkably, lysine and valine, as the most deficient EAAs, in particular, were compensated with increasing BPF level. The major NEAAs found in BPF were glutamic acid, arginine, and aspartic acid.

The percentage of amino acids and calculated biological efficiency, essential amino acid index (EAAI), calculated protein efficiency ratio (PER), and requirement index of different age groups are presented in Table 6. Branched-chain amino acids (BCAAs) are a group of three essential amino acids: leucine, isoleucine, and valine, which commonly boost muscle building, enhance exercise performance, and reduce fatigue. Substituting WF with 5, 10, and 15% BPF increased the BCAAs content by 0.70, 1.79, and 2.14%, respectively. The basic amino acids (BAAs) content, such as that of lysine, arginine, and histidine, was increased up to 5.58, 10.93, and 15.88% with 5, 10, and 15% of BPF, respectively. Total uncharged polar AAs (glycine, serine, threonine, tyrosine, and cysteine) increased by 0.61, 1.58, and 1.85% in WF + 5% BPF, WF + 10% BPF, and WF + 15% BPF, respectively.

In the same context, the calculated PER by relevant equations showed that PER values underwent a major improvement during the addition of BPF to WF. The values increased from 19.88 to 20.21, 20.60, and 20.85 with 5, 10, and 15% of BPF, respectively. This indicated that increasing BPF improved the biological availability of cake protein. Calculated biological and EAAI values increased from 20.29 to 21.23, 22.48, and 23.11 and from 44.68 to 45.20, 45.86, and 46.19 with 5, 10, and 15% of BPF, respectively. As the cake is preferred and served to various age groups, the requirement index depending on World health organization (WHO, 2007) was calculated, and results are presented in Table 6. Obviously, incorporating BPF in cake formulas improved the nutritious value and gradually increased the requirement index in all age groups.

### 3.5. Effect of WF Substitution with BPF on Rheological and Thermo-Mechanical Parameters Examined by MIXOLAB

**Figure 1:**
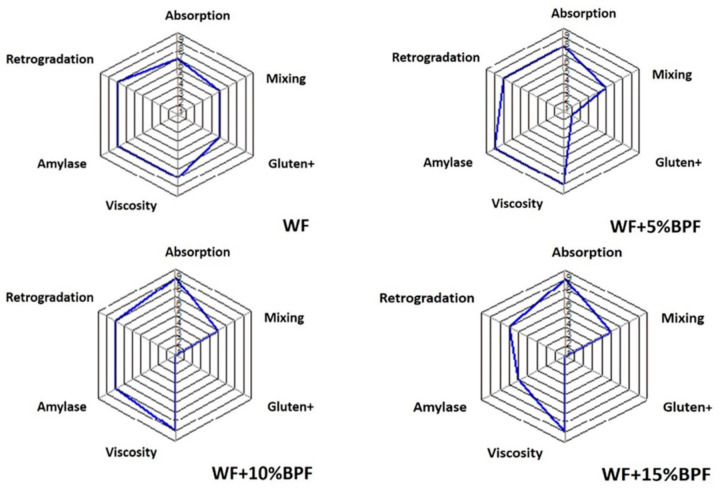
The Mixolab standard profile of WF 72% and substituted WF with BPF (*A. digitate*) at different substitution levels 5% (WF + 5% BPF), 10% (WF + 10% BPF), and 15% (WF + 15% BPF).

**Figure 2:**
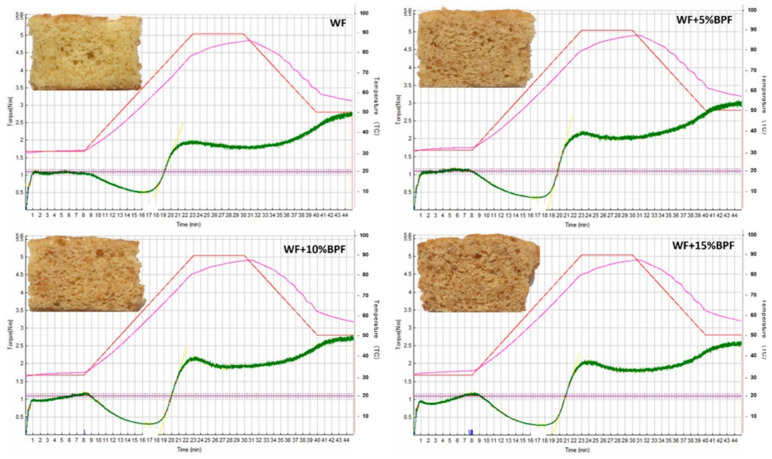
The thermo-mechanical and rheological properties of WF 72% and substituted WF with BPF (*A. digitate*) at different substitution levels, 5% (WF + 5% BPF), 10% (WF + 10% BPF), and 15% (WF + 15% BPF).

In the Mixolab analysis, five parameters must be considered; (C1): maximum torque of the initial mixing phase, (C2): protein weakening, (C3): starch gelatinization, (C4): physical breakdown of gelatinized starch granules, and (C5): starch retrogradation, as explained by Kahraman et al. [57]. The impact of WF substitution by BPF on the absorption, mixing, gluten, viscosity, amylase, and retrogradation was investigated, and data are shown in Table 7 and Figure 1 and Figure 2. Partial substitution of WF with BPF has changed the dough properties as recorded in their Mixolab profiles. According to obtained results from Mixolab data sheets, the addition of BPF to WF led to a noticeable effect on the rheological properties of WF, which affected the dough behavior and cake characteristics. The water absorption rate showed a significant improvement with the increase of BPF addition level. The absorption and viscosity were increased gradually with increasing the BPF levels, particularly, with increasing substitution level. Adding 15% of BPF increased water absorption by up to 5%. Adding BPF reduced gluten network stability, but it was exponentially remarked when BPF added up to 15%.

However, mixing and retrogradation were not drastically changed, up to 15% for mixing and up to 10% for retrogradation (Figure 1 and Figure 2). Incorporation of 5% BPF was desirable to give the appropriate texture and ability of cake to retain gases and reach the appropriate shape and volume. However, increasing BPF up to 10–15% caused a slight weakness in the gluten network, which may be needed to attain dough and product properties when strong flour is used. Conversely, substituting WF with 5%, 10%, and 15% BPF reduced the stability by 11.78%, 39.34%, and 49.55%, respectively. As shown in Figure 1 and Figure 2, it looks like there are variations in the thermo-mechanical and rheological parameters of substituted WF at a temperature of 30 °C based on the BPF substitution level. No significant changes were observed in starch parameters when 5–10% of BPF was added. As for C2, an obvious difference was observed among all cake formulas. On the contrary, WF has recorded the highest torque (0.504 Nm) for C2 compared with other formulas. For C3, C4, and C5, adding 5% of BPF increased the torque value, then a sequent decrease was recorded with 10 and 15% BPF. Combining the Mixolab parameters and Mixolab profile, results indicated that 5–10% BPF kept all characters concerning viscosity and retrogradation indices close to those of the WF cake, but with 15% BPF, a significant recede observed in the retrogradation index (Figure 1).

### 3.6. The Organoleptic Properties of Cake Made from WF and Substituted WF with BPF

The impact of substituted WF with BPF used to make the cake on organoleptic properties was evaluated, and data are shown in Table 8. The panelists were asked to judge cake parameters include crust color, odor, taste, crumb cells, and texture as well as overall acceptability. Significant differences (*p* < 0.05) were observed between WF-cake and WF + BPF-cakes in all organoleptic characteristics with increasing BPF subistution levels.

Increasing BPF up to 15% affected the organoleptic properties, but the panelists still considered the organoleptic characteristics as acceptable. For example, the greater and smaller overall acceptability was observed for WF and WF + 15% BPF cakes to be 86.33 and 73.33%, respectively. Crust color, odor, and taste differed significantly (*p* < 0.05) with a higher level of BPF, which may reflect the distinguished color, odor, and taste of BPF. Regarding the crumb cells properties, the WF + 15% BPF cake recorded a significant difference (*p* < 0.05) in color, uniformity, size, and thickness properties. WF + 5% BPF cake was comparable to WF cake, and significant differences (*p* < 0.05) in the texture properties were observed between WF cake and BPF cakes with high substitution levels. Likewise, WF + 15% BPF cake was the lowest formula in softness, tenderness, and moistness. The data in Figure 2 illustrate that the addition of BPF at 5 or 10% did not dramatically influenced the visual organoleptic properties when compared with WF cake.

## 4. Discussion

The cake is considered one of the most widely consumed bakery products in the world, with an estimated global cake market size of USD 42.94 billion in 2019, which is expected to rise at a compound annual growth rate of 3.3% from 2020 to 2027 [58]. It is well known that cakes are based mainly on WF in manufacturing with various food ingredients. 

Interestingly, cakes are essential for celebrating various types of occasions, favored for children, serving consumers with wide age-grouping, used as a snack between diets, or as desserts post meals during dine-out. However, to help customers fulfill their nutritional requirements, food fortification is a helpful and valuable technique [38,39,40,59]. Fortification is not materializing as one single issue to that purpose, but also coexists for dietary diversification and supplementation approaches. It also provides a relatively easy solution to enrich micronutrient intakes in traditional dietary patterns and ensured that food diversification can be maintained [60,61]. Consequently, substitution levels were proposed in this experiment to provide a substantial amount of EAAs, minerals, and even fiber to complement the intake of some deficient nutrients in the cake.

In the current study, incorporating BPF in WF to be used in cake manufacturing increased the macro- and microelements [12,30,62]. Compaoré et al. [62] confirmed that BPF has a good potential in macro and micronutrient content and valorizing it can effectively be used to fortify staple food, particularly for children, and contribute to eradicate malnutrition due to micronutrients deficiencies. Our results remarked that substituting 5–15% of WF with BPF significantly increased total ash [63] and fiber content without significant changes in crude protein, available carbohydrates contents, and energy values of prepared flour mixtures. This may be due to the BPF containing considerable amounts of minerals [63] and fiber [12,13,62], and being a good source of EAAs [9] and carbohydrates [63]. Current results in Table 2 are very close to those of Nour et al. [64], Osman [9], and are additionally confirmed by Fagbohun et al. [63], who indicated that BPF contains 10.2, 7.67, 0.4, 5.7, 12.16, 73.87% and 307.6 kcal g^−1^ for moisture, ash, fat, crude fiber, crude protein, carbohydrate, and metabolizable energy.

Regrading to mineral analysis, the results in Table 3 demonstrate that K and Fe were the most abundant macro- and microelements in BPF when compared with WF, respectively. Na presented low content in BPF and high content in WF. These observations are in accordance with previous studies of Osman [9], Compaoré et al. [62], and Adubiaro et al. [65]. The obtained results demonstrated that BPF-enriched WF in the range of 5–15% could represent modified macro- and microelements profiles with significant increases in Ca, K, P, Mg, Zn, Fe, and Cu content and a significant decrease in Na content to improve the nutritional profile and desired health attributes [31]. Recently, Debelo et al. [30] recommended that the incorporation of BPF in the range of 5–15% in composite pearl millet porridges modified the bioaccessibility of carotenoids and produced rich mineral products.

Interestingly, a remarkable incremental trend in TPC and AOA in BPF-enriched WF was observed (Table 4). This may be due to the rich content of bioactive compounds and phytochemicals in BPF, which was increased with increasing BPF levels. The substitution of WF with BPF into cake flour formulas improved the AOA by more than 50 and 60%, assessed by DPPH and ABTS scavenging assays, respectively. The antioxidant capacity was probably due to the presence of phenolic compounds and ascorbic acid [16,18,32,63]. The AOA could improve the bakery products’ shelf-life stability and delay oil oxidation, particularly in cake products. These results agree with [47,66,67]. Recently, Debelo et al. [30] recommended that the incorporation of BPF in the range of 5–15% in composite pearl millet porridges modified the bioaccessibility of carotenoids and produced rich minerals products.

Concerning the amino acid composition, our results showed that the addition of BPF verified a change in the amino acid composition in the prepared composite flour, in particular for EAAs (Table 5 and Table 6). Amino acid profile in BPF demonstrated rich EAA and NEAA content, surpassing the WF profile, which made it useful in raising the AA content, as expected according to various studies that confirmed valuable AA content of BPF [9,14,17]. The analyzed AA profile in our study was closely associated that of with Osman [9], who stated that the sulfur-containing amino acids were present in low content, while the predominant EAAs in *A. digitata* were leucine and phenylalanine and predominant NEAAs were glutamic acid and tyrosine in seed and fruit pulp, respectively. Our results noticed that the predominant EAA in *A. digitata* pulp flour was leucine and the predominant NEAA was glutamic acid. Similarly, Busson et al. [14] observed that *A. digitata* fruit pulp had the highest glutamic acid content, with lower content of the sulfur-containing amino acids. In the same context, the amino acid content of *A. digitata* revealed glutamic acid was the most abundant amino acid, followed by aspartic acid, lysine, leucine, proline, alanine, and valine [17,28,68]. Remarkably, lysine, as the most deficient EAA in WF, was compensated by 5.9, 11.4, and 16.7% when BPF was incorporated at 5%, 10%, and 15%, respectively, as a result of high lysine content in BPF, as previously confirmed [9,17,28].

Interestingly, our results showed that the addition of BPF determined a considerable change in the biological efficiency, EAAI, PER, and requirement index of different age groups, as presented in Table 6. BCAAs are a group of three essential amino acids: leucine, isoleucine, and valine, which commonly booste muscle building, enhance exercise performance, and reduce fatigue [69]. The incorporation of BPF increased the BCAA content in a dose-dependent manner, and increasing the substitution level could increase the BCAAs, resulting in a cake with high nutritious and biological value. The best enrichment was noticed in BAA content, such as that of lysine, arginine, and histidine, which associatively increased up to 5.58%, 10.93%, and 15.88% with increasing BPF level. The basic amino acids are highly associated with increasing protein bioactivity [70] and possess antioxidant and antimicrobial activities [71].

Similarly, total uncharged polar AAs (glycine, serine, threonine, tyrosine, and cysteine) have shown an increase in a dose-dependent manner. Singh and Sogi [72] stated that increasing uncharged polar AAs might be responsible for increasing protein solubility. PER is the easiest method of assessing the quality of proteins [73]. The calculated PER indicated major improvement during adding BPF to WF in a dose-dependent manner, which could improve the biological availability of presented protein content in cakes [7,17]. Similarly, incorporating BPF with WF exuded noticeable increases in calculated biological and EAAI values, which may be due to the rich content of EAAs in BPF [9]. According to WHO, 2007 amino acid requirements, incorporating BPF in cake formulas improved the nutritious value and gradually increased the efficacy of prepared WF + BPF composite mixtures to cover the protein requirements of different age groups.

The Mixolab analysis is a globally standardized test primarily intended to measure the rheological properties of the dough and thereby ensure a smooth manufacturing process and the quality of finished bakery products. The quality of a cereal-based product is appreciated not only through its characteristics of taste and nutrition but also through its physical characteristics. These physical characteristics depend largely on the quantity and functionality of the protein, starch, and enzymes that make up flour [74]. To measure the combined effect of WF and BPF, the behavior during kneading of WF + BPF dough was subjected to a Mixolab analysis to check the quality and regularity of the flours in a single test [57]. The incorporation of BPF increased the water absorption capacity, which indicates the quantity of product that can be produced from a given quantity of flour, which is an important economic factor. A gradual increase in water absorption, which was up to 10% in WF + 15% BPF, was possibly due to the rich content of pectin, minerals, fiber, and polysaccharides in BPF [9,62,68]. According to sundry previous publications, the water absorption rate directly affects the amount of profit, the shelf-life of the product, and the smoothness of technological processes during the manufacturing process [75,76]. The behavior of dough during kneading helps to ensure that the raw material is compatible with industrial processing. Remarkably, increasing BPF level reduced the stability of dough in dose-dependent manner during kneading. However, the major factors to decrease gluten stability are sugars and pectin in BPF, which cause weaknesses in the gluten network [74]. Sometimes this change is seen as undesirable, but in our study, this change is somewhat desirable to give the appropriate texture and the ability of the cake to retain gases to reach the appropriate shape and volume [75]. In our study, WF containing BPF showed curved increases in dough viscosity associated with rising α-amylase activity when BPF was added up to 10%. Subsequently, adding 15% of BPF dramatically affected the α-amylase activity, possibly due to the presence of thermostable proteinaceous α-amylase inhibitors [77]. The changing in the viscosity of the dough as its temperature increases gives indications about the internal structure of the finished product. Interestingly, the viscosity and stability at 90 °C provide information about the amylase activity, which has an impact on the color of the finished product. Finally, the cooling phase indicates the rate of starch retrogradation, which is a direct relationship with the shelf-life. Concerning viscosity index and retrogradation index, with the highest addition (15%) of BPF, a significant recede was observed in retrogradation index, which relates closely with product properties after production and determines the shelf-life of the product. Indeed, to the best of our knowledge, no work about the thermo-mechanical and rheological parameters of WF substituted with BPF was found, and the most important findings were agreed by Kahraman et al. [57]. Regardless of changes in gluten index, which modifies the flour mixture for satisfying cake making, the addition of BPF at 5% and 10% to cake product kept all characteristics close to those of the WF cake sample.

Sensory evaluation indicated significant differences between WF cake and WF + BPF cakes in all organoleptic characteristics as a result of substituting with a high level of BPF with distinguished taste. However, no significant changes in all organoleptic parameters could be observed, even a positive improvement in taste using 5% BPF was recorded, and it is encouraging that adding BPF at a low level can enhance some of the organoleptic properties [33]. Conclusively, 5–10% of BPF did not drastically affect the organoleptic characteristics of WF + BPF cake, even adding 15% was still acceptable, but using high substituting levels will not only affect the organoleptic characteristics slightly but will also influence the rheological properties [42,77]. On the contrary, Mounjouenpou et al. [78] stated that the incorporation of BPF at 20% improved the sensory and nutritional qualities of rice cookies. 

## 5. Conclusions

A successful and pioneering formulation of cake production with BPF was developed. Incorporating BPF significantly increased the total ash and crude fiber content in composite flour in a level-dependent manner, while moisture, crude fat, crude protein, available carbohydrate content, and energy values were not significantly changed. Interestingly, macroelements such as Ca, K, and P were significantly increased, while Na was significantly decreased, whereas Mg content was not significantly changed. Similarly, microelements such as Zn, Fe, and Cu have significantly increased associatively with increasing BPF. Significant increases in TPC and AOA were highlighted by increasing BPF level. The BPF could possess an AOA to boost shelf-life stability, decrease oil rancidity, and improve staling. The BV, EAAI, and PER as well as RI were positively improved in WF + BPF. Adding BPF up to 10% not only improved the water absorption, α-amylase activity, and viscosity but also caused a slight weakness in the gluten network, which produces a composite flour suitable for cake making. The substitution of WF with 5–10% is recommended to make an acceptable cake by consumers. Interestingly, this study revealed that fortification with BPF as a nutritious and unconventional material by up to 5% improved the nutrition quality without adverse effects on rheological, technological, and organoleptic characteristics, and provided economic, commercial, and health benefits. Further studies concerning keeping stability and physicochemical properties are needed. 

## Figures and Tables

**Table 1:** Raw ingredients (g) of processed cake formulas substituted with BPF (*A. digitate*).

Ingredients	WF	WF + 5% BPF	WF + 10% BPF	WF + 15% BPF
Soft wheat flour (72% extraction)	250.0	237.5	225	212.5
Baobab pulp flour (BPF)	-	12.5	25	37.5
Sucrose powder	125.0	125.0	125.0	125.0
Salt	3.50	3.50	3.50	3.50
Skimmed milk powder	25.0	25.0	25.0	25.0
Shortening	53.50	53.50	53.50	53.50
Fresh whole egg	110.0	110.0	110.0	110.0
Baking powder	12.50	12.50	12.50	12.50
Vanilla	2.0	2.0	2.0	2.0

WF: Wheat flour, WF + 5% BPF: WF substituted with 5%, WF + 10% BPF: WF substituted with 10% BFP, WF + 15%: WF substituted with 15%.

**Table 2:** The chemical composition of WF and various substituted WF with BPF (*A. digitate*).

Chemical Composition	Cake Formulas				
	BPF	WF	WF + 5% BPF	WF + 10% BPF	WF + 15% BPF
Moisture	13.28 ± 0.26 ^a^	11.70 ± 0.59 ^b^	11.80 ± 0.35 ^b^	11.84 ± 0.47 ^b^	11.97 ± 0.42 ^b^
Crud protein *	12.54 ± 0.14 ^a^	12.93 ± 0.65 ^a^	12.93 ± 0.39 ^a^	12.87 ± 0.51 ^a^	12.90 ± 0.45 ^a^
Ash *	6.52 ± 0.05 ^a^	0.83 ± 0.04 ^e^	1.13 ± 0.03 ^d^	1.38 ± 0.06 ^c^	1.71 ± 0.06 ^b^
Crude fat *	0.45 ± 0.05 ^c^	0.95 ± 0.05 ^a^	0.95 ± 0.03 ^a^	0.88 ± 0.04 ^b^	0.91 ± 0.03 ^a^
Crude fiber *	4.65 ± 0.23 ^a^	0.88 ± 0.04 ^e^	1.09 ± 0.03 ^d^	1.24 ± 0.05 ^c^	1.48 ± 0.05 ^b^
Available carbohydrate *	75.84 ± 0.15 ^b^	84.41 ± 4.22 ^a^	84.00 ± 2.52 ^a^	83.53 ± 3.34 ^a^	83.15 ± 2.91 ^a^
Energy value (Kcal 100 g^−1^)	357.57 ± 9.21 ^a^	351.75 ± 17.59 ^a^	349.86 ± 10.50 ^a^	347.30 ± 13.89 ^a^	345.75 ± 12.10 ^a^

*: The data was manipulated on a dry weight basis, ^a–c^: means with the same letter in the same row are not statistically significant.

**Table 3:** Mineral content of WF and various substituted WF with BPF (*A. digitate*).

Minerals (mg 100 g^−1^) *	Cake Formulas				
	BPF	WF	WF + 5% BPF	WF + 10% BPF	WF + 15% BPF
Macroelements	-	-	-	-	-
Na	36.02 ± 1.08 ^e^	186.54 ± 4.46 ^a^	177.51 ± 3.04 ^b^	171.85 ± 3.73 ^c^	162.10 ± 2.87 ^b^
Ca	237.03 ± 7.11 ^a^	35.89 ± 1.44 ^e^	47.96 ± 1.63 ^d^	58.37 ± 2.28 ^c^	65.70 ± 2.50 ^b^
K	987.51 ± 29.63 ^a^	165.47 ± 6.62 ^e^	214.79 ± 7.30 ^d^	257.55 ± 10.04 ^c^	287.12 ± 7.16 ^b^
P	124.08 ± 3.72 ^a^	86.15 ± 3.45 ^b^	88.43 ± 3.01 ^b^	91.18 ± 3.56 ^b^	90.98 ± 3.46 ^b^
Mg	102.01 ± 3.06 ^a^	84.54 ± 3.10 ^b^	85.59 ± 2.37 ^b^	87.31 ± 2.70 ^b^	86.32 ± 1.89 ^b^
Microelements	-	-	-	-	-
Zn	2.04 ± 0.06 ^a^	0.66 ± 0.05 ^d^	0.74 ± 0.03 ^c^	0.82 ± 0.05 ^bc^	0.86 ± 0.07 ^b^
Fe	9.03 ± 0.27 ^a^	1.21 ± 0.04 ^e^	1.68 ± 0.05 ^d^	2.08 ± 0.07 ^c^	2.37 ± 0.08 ^b^
Cu	1.53 ± 0.05 ^a^	0.28 ± 0.01 ^d^	0.35 ± 0.03 ^c^	0.42 ± 0.02 ^b^	0.46 ± 0.04 ^b^

*: The data was manipulated on a dry weight basis, ^a–e^: means with the same letter in the same raw are not statistically significant.

**Table 4:** The total phenolic content and antioxidant activity of WF and various substituted WF cake with BPF (*A. digitate*).

Organoleptic Attributes	Cake Formulas			
	WF	WF + 5% BPF	WF + 10% BPF	WF + 15% BPF
Total phenolic compounds (mg GAE g^−1 dw^)	3.15 ± 0.13 ^e^	3.55 ± 0.18 ^d^	3.96 ± 0.10 ^c^	4.36 ± 0.20 ^b^
Antioxidant activity, DPPH (mg TE g^−1 dw^)	5.47 ± 0.22 ^e^	6.45 ± 0.32 ^d^	7.44 ± 0.21 ^c^	8.42 ± 0.38 ^b^
Antioxidant activity, ABTS (mg TE g^−1 dw^)	6.48 ± 0.26 ^e^	7.89 ± 0.49 ^d^	9.31 ± 0.31 ^c^	10.72 ± 0.49 ^b^

^a–e^: means with the same letter in the same row are not statistically significant.

**Table 5:** Amino acid composition (mg g^−1^ protein) of WF and different substituted WF with BPF (*A. digitate*).

Amino Acid	Amino Acid (AA) mg/g Protein				
	BPF	WF	WF + 5% BPF	WF + 10% BPF	WF + 15% BPF
**EAA**					
Therionine	30.30	25.68	25.88	26.19	26.30
Valine	50.24	39.75	40.25	40.88	41.23
Isoleucine	35.89	31.86	32.03	32.32	32.38
Leucine	63.80	57.54	57.78	58.27	58.32
Phenylalanine	49.44	45.94	46.02	46.39	46.32
Lysine	52.63	24.90	26.37	27.74	29.05
Hisitidine	23.92	21.81	21.89	22.07	22.08
Cystine	24.72	18.72	19.03	19.35	19.60
Methonine	19.14	20.26	20.17	20.18	20.03
**NEAA**					
Aspartic	90.11	48.26	50.45	52.61	54.53
Serine	46.25	37.28	37.70	38.25	38.53
Glutamic	240.03	229.54	229.73	230.96	230.41
Proline	38.28	120.65	115.97	112.40	107.62
Glycine	43.06	36.50	36.80	37.23	37.39
Alanine	48.64	31.86	32.72	33.63	34.34
Tyrosine	29.51	34.18	33.87	33.76	33.36
Argenine	107.66	42.85	46.29	49.54	52.65
EAAs	350.08	286.47	289.42	293.38	295.30
NEAAs	643.54	581.13	583.54	588.37	588.83
TAA	993.62	867.59	872.96	881.75	884.13

EAA: essential amino acids, NEAA: nonessential amino acid, TAA: total amino acids.

**Table 6:** Percentage of amino acids and calculated biological efficiency, essential amino acid index, estimated protein efficiency ratio, and requirement index of different age groups.

Parameters	BPF	WF	WF + 5% BPF	WF + 10% BPF	WF + 15% BPF
Total BCAAs (mg/g protein)	149.92	129.16	130.07	131.47	131.93
Total BAAs (mg/g protein)	184.21	89.56	94.55	99.35	103.78
Total uncharged polar AAs	173.84	152.36	153.28	154.77	155.18
PER	26.61	19.88	20.21	20.60	20.85
BV	44.54	20.29	21.23	22.48	23.11
EAAI	54.34	44.68	45.20	45.86	46.19
Requirement index (Infants)	116.41	95.72	96.84	98.24	98.96
Requirement index (Preschool child)	126.43	103.96	105.18	106.70	107.48
Requirement index (Schoolchild)	138.35	113.76	115.09	116.76	117.62
Requirement index (Adult)	145.50	119.63	121.04	122.79	123.69

BCAAs: branched-chain amino acids, BAAs: basic amino acids, BV: calculated biological value, EAAI; essential amino acid index, PER: calculated protein efficiency ratio.

**Table 7:** Water absorption and stability of WF and substituted WF with BPF (*A. digitate*).

Composite Flour *	Mixolab Parameters					
	Water Absorption (% b14) **	Stability (min)	C2 (Nm)	C3 (Nm)	C4 (Nm)	C5 (Nm)
WF 72%	59.1 ± 0.9 ^c^	8.82 ± 0.41 ^a^	0.504 ± 0.012 ^a^	1.942 ± 0.034 ^b^	1.780 ± 0.057 ^c^	2.750 ± 0.035 ^b^
WF + 5%BPF	60.3 ± 0.7 ^c^	7.78 ± 0.52 ^a^	0.348 ± 0.024 ^b^	2.157 ± 0.047 ^a^	2.006 ± 0.078 ^a^	2.978 ± 0.027 ^a^
WF + 10%BPF	62.4 ± 0.6 ^b^	5.35 ± 0.19 ^b^	0.299 ± 0.017 ^c^	2.148 ± 0.057 ^a^	1.903 ± 0.024 ^b^	2.711 ± 0.045 ^b^
WF + 15%BPF	64.6 ± 0.5 ^a^	4.45 ± 0.67 ^c^	0.275 ± 0.032 ^c^	2.040 ± 0.071 ^a^	1.803 ± 0.057 ^c^	2.570 ± 0.052 ^c^

*: These mixtures were composed of WF substituted by BPF at different levels, **: these data were basically calculated on 14% moisture content in WF, ^a–c^: means with the same letter in the same column are not statistically significant.

**Table 8:** Organoleptic properties of WF and substituted WF with BPF (*A. digitate*).

Organoleptic Attributes		Cake Formulas			
		WF	WF + 5% BPF	WF + 10% BPF	WF + 15% BPF
Crust color (10)		8.83 ± 0.40 ^a^	8.33 ± 0.16 ^a^	7.50 ± 0.21 ^b^	7.08 ± 0.50 ^b^
Odor (10)		9.00 ± 0.30 ^a^	8.50 ± 0.29 ^a^	8.00 ± 0.33 ^a,b^	8.17 ± 0.44 ^a,b^
Taste (10)		8.25 ± 0.88 ^a^	8.92 ± 0.25 ^a^	8.08 ± 0.25 ^a,b^	7.83 ± 0.35 ^b^
Crumb cells (40)	Thickness (10)	8.75 ± 0.38 ^a^	8.58 ± 0.21 ^a^	7.92 ± 0.28 ^b,c^	7.50 ± 0.42 ^b,c^
	Size (10)	9.00 ± 0.30 ^a^	8.42 ± 0.28 ^a^	7.50 ± 0.37 ^b^	6.42 ± 0.39 ^c^
	Uniformity (10)	8.75 ± 0.33 ^a^	7.92 ± 0.28 ^b^	7.17 ± 0.30 ^c^	6.50 ± 0.37 ^d^
	Color (10)	8.25 ± 0.51 ^a^	8.00 ± 0.19 ^a^	7.5 ± 0.37 ^a,b^	6.75 ± 0.47 ^b,c^
Texture (30)	Softness (10)	8.75 ± 0.41 ^a^	8.42 ± 0.25 ^a,b^	8.00 ± 0.30 ^a,b^	7.67 ± 0.43 ^b,c^
	Tenderness (10)	9.00 ± 0.30 ^a^	8.17 ± 0.33 ^b^	7.83 ± 0.30 ^b^	7.42 ± 0.34 ^b,c^
	Moistness (10)	8.75 ± 0.38 ^a^	8.17 ± 0.33 ^a,b^	7.92 ± 0.37 ^b^	7.58 ± 0.53 ^b^
Overall acceptability (100)		86.33 ± 3.41 ^a^	82.92 ± 1.76 ^a^	77.67 ± 1.61 ^b^	73.33 ± 2.82 ^b^

^a–c^: Means with the same letter in the same raw are not significantly different (*p >* 0.05).

## Data Availability

Not applicable.
